# Combination of 2D/3D Ligand-Based Similarity Search in Rapid Virtual Screening from Multimillion Compound Repositories. Selection and Biological Evaluation of Potential PDE4 and PDE5 Inhibitors

**DOI:** 10.3390/molecules19067008

**Published:** 2014-05-28

**Authors:** Krisztina Dobi, István Hajdú, Beáta Flachner, Gabriella Fabó, Mária Szaszkó, Melinda Bognár, Csaba Magyar, István Simon, Dániel Szisz, Zsolt Lőrincz, Sándor Cseh, György Dormán

**Affiliations:** 1 Targetex Ltd., Kápolna köz 4/a., Dunakeszi 2120, Hungary; E-Mails: dobi@targetex.com (K.D.); hajdu@targetex.com (I.H.); flachner@targetex.com (B.F.); fabo@targetex.com (G.F.); szaszko@targetex.com (M.S.); bognar@targetex.com (M.B.); lorincz@targetex.com (Z.L.); cseh@targetex.com (S.C.); 2 Institute of Enzymology, Natural Sciences Research Center, Hungarian Academy of Sciences, Magyar Tudósok körútja 2, Budapest 1117, Hungary; E-Mails: magyar.csaba@ttk.mta.hu (C.M.); simon.istvan@ttk.mta.hu (I.S.); 3 Chemaxon Ltd., Záhony u. 7, Budapest 1038, Hungary; E-Mail: dszisz@chemaxon.com

**Keywords:** 2D similarity, 3D similarity, ligand-based virtual screening, focused library, phosphodiesterase 4, phosphodiesterase 5, flexible alignment

## Abstract

Rapid *in silico* selection of target focused libraries from commercial repositories is an attractive and cost effective approach. If structures of active compounds are available rapid 2D similarity search can be performed on multimillion compound databases but the generated library requires further focusing by various 2D/3D chemoinformatics tools. We report here a combination of the 2D approach with a ligand-based 3D method (Screen3D) which applies flexible matching to align reference and target compounds in a dynamic manner and thus to assess their structural and conformational similarity. In the first case study we compared the 2D and 3D similarity scores on an existing dataset derived from the biological evaluation of a PDE5 focused library. Based on the obtained similarity metrices a fusion score was proposed. The fusion score was applied to refine the 2D similarity search in a second case study where we aimed at selecting and evaluating a PDE4B focused library. The application of this fused 2D/3D similarity measure led to an increase of the hit rate from 8.5% (1st round, 47% inhibition at 10 µM) to 28.5% (2nd round at 50% inhibition at 10 µM) and the best two hits had 53 nM inhibitory activities.

## 1. Introduction

In the recent years we have witnessed an explosion in the number of compounds available for biological screening. For example, the ZINC database contains approximately 30 million molecules that are commercially available from various sources [1]. Furthermore, several databases provide access to biological activity data for small molecules that can serve as input data for model building and virtual screening. For example, PubChem [2] contains 119,958,113 reported compounds together with 717,473 bioassay counts; ChEMBL [3] collects 1,292,300 compounds with 11,000,000 activities for 9000 targets from 45,000 publications; Binding_DB (2013) [4] reports 773,900 compounds with 1,010,714 binding data for 6910 protein targets as public databases. Commercial databases that monitor the progress of drug candidates are Pharmaprojects [5], with searchable profiles for over 50,000 drugs and drug candidates; Integrity database [6] which is a multidisciplinary database supporting the drug research and development. Such large data collections could be exploited in *in silico* screening particularly if limited biological screening capacity is available for researchers. Furthermore, in many cases virtual screening and *in vitro* HTS are used as complementary tools [7].

Random screening of a discovery library often results in a poor hit rate (≤0.1%) [8] while the hit rate of *in silico* focused libraries could reach 1%. Furthermore, by means of the use of focused libraries the synthesis, repository management and screening costs could be reduced [9]. Up to present various ligand-based (LBVS) and structure-based virtual screening (SBVS) methods [10] have been reported for selecting focused libraries from compounds repositories [11].

Even though computational efficiency has improved significantly in the recent years, more reliable scoring and tedious post-processing is required in structure-based VS methods. Therefore, ligand—based VS approaches have become popular including: pharmacophore models, neural networks, discrimination of known active and known inactive molecules *etc*. Ligand-based similarity search methods using 2D fingerprints are very effective and widely used approaches that apply the old similarity principle: “structurally similar molecules are likely to have similar properties” [12,13].

2D similarity search is certainly the method of choice when numerous active reference compounds and multimillion compound databases are available [14]. Similarity is generally expressed in Tanimoto coefficients [15].

The search results could be focused by property–based filtering taking into consideration of the physico-chemical parameter space of the reference compounds. On the other hand it is claimed that “similarity-based compound ranking is an insufficient indicator of the biological activity” [9].

3D target or ligand-based approaches could help for refining the 2D virtual screening results and increasing further the hit rate. For example 2D similarity search results could be ranked according to docking scores if proper 3D models are available. One of the major challenges of docking is protein flexibility [16] and the adaption of the proper conformation is a critical element in ligand binding. Either multiple static binding conformations can be considered to overcome the problem or docking should allow molecular dynamics simulations [17]. The situation is more complicated, if water molecules participate in the binding. The above methods can be also target specific and these methods do not work effectively with all protein types.

All these arguments highlight the fact why 2D similarity methods still enjoy high popularity and also explain the high demand for rapid, innovative solutions in terms of 3D ligand–based methods.

Ligand-based approaches do not take the target structure directly into account. These techniques are based on the assumption that compounds with similar topology have similar biological activities.

The possible binding features of small molecules can be assessed by their conformational flexibility and shape. The conformations are created on-the-fly, and the statistical representation of all possible conformations could be viewed as a 3D structure. During flexible alignment (such as applied in Screen3D software) dynamic 3D structures can be compared and their similarity or dissimilarity can be determined and expressed in 3D similarity measures. Flexible alignment methods do not require a pre-defined set of initial conformers to sample the conformational space of the molecules. During the alignment procedure atom-type information would be capable of generating alignments where patterns with similar binding character are oriented in a similar fashion in the known active and the candidate structures. This provides a more realistic picture of the potential bioactive similarity of the molecules.

In the present study our objective was to evaluate the utility of the 3D flexible alignment approach in similarity-based focused library generation. We have chosen two important members of the phosphodiesterase family, PDE5 and PDE4 as biological targets for this study. Phosphodiesterase (PDE) enzymes are implicated in various physiological processes mediated by cAMP or cGMP. PDE inhibitors, which block the degradation of the above second messengers regulated by the enzymes, are potential therapeutic agents in various pulmonary, cardiovascular diseases as well as in CNS disorders. PDE5 inhibitors are used in the treatment of erectile dysfunction, as the first effective oral medication, as well as for the therapy of pulmonary hypertension.

PDE4 inhibitors are known to possess long-term memory-improving, neuroprotective and particularly anti-inflammatory effects that are useful in the therapy of chronic obstructive pulmonary disease (COPD), asthma and rheumatoid arthritis [18].

In a previous screening campaign we generated a PDE5 focused library where the compounds were selected exclusively by 2D similarity search methods and screened in *in vitro* assays [19]. The dataset generated during the study served as an ideal starting point to analyze and correlate 2D and 3D similarities as well as giving us a hint how to combine (fuse) the 2D and 3D similarity measures in further applications, namely, selecting PDE4 inhibitors from large vendor compound libraries.

In the present paper we report: (a) 2D/3D similarity analysis of a previous phosphodiesterase 5 (PDE5) screening campaign; (b) combination of 2D and 3D similarity search for selecting a hit validation focused library in a PDE4 screening campaign. Similarities are expressed in 2D (T2D) and 3D (T3D) Tanimoto coeffiecients. As starting points we collected 44 structurally different active PDE5 inhibitors and 44 PDE4 inhibitors representing from literature and available databases (see Supplementary Material).

## 2. Results and Discussion

### 2.1. Case Study 1: 2D/3D Analysis of Available Data from PDE5 Screening

First round screening: The 2D selection strategy for the generation of a PDE5 focused library generation was reported earlier [19]. We used such dataset to investigate the correlation between the 2D and 3D similarities and learn how to apply flexible alignment (Screen3D software) more efficiently in further *in silico* screening campaigns. We involved five hit compounds (IC_50_ < 10 µM, Table 1) in our analysis reported that were selected based on the structure of three known PDE5 inhibitors as reference or seed compounds (#13, #18, #44, see Table 1—column “Seeds”).

**Table 1:** First round screening hits as potential PDE5 inhibitors with T2D and T3D values.

ID	Hits	IC_50_ (µM)	T2D	T3D	Seeds	Seed ID
**1**	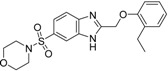	3.65	0.63	0.47	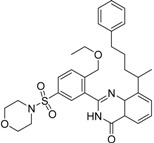	13
**2**	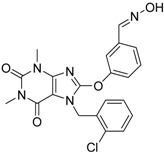	1.99	0.61	0.38	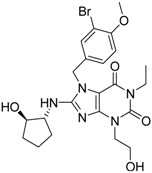	18
**3**	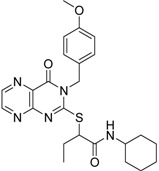	7.24	0.61	0.31	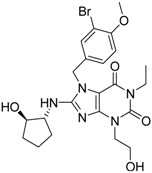	18
**4**	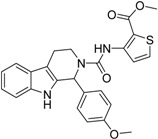	0.19	0.69	0.35	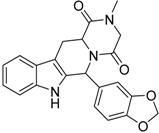	44
**5**	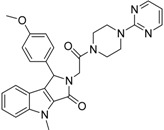	6.74	0.67	0.38	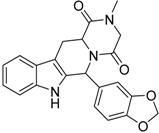	44

The 3D similarities were calculated by the Screen3D software and correlated with the 2D (T2D) values. While the T2D values were over 0.60 (that was the criteria of the selection), the 3D Tanimoto scores (T3D) were much lower and were often just above 0.3. The significant differences in the 2D (T2D > 0.60), and 3D Tanimoto scores (T3D > 0.3) revealed that even though the compounds selected by 2D methods had similar molecular architectures, they are rather different in terms of shape and conformational flexibility. Such 3D dissimilarity is assumed to represent much different binding characteristics.

**Figure 1:**
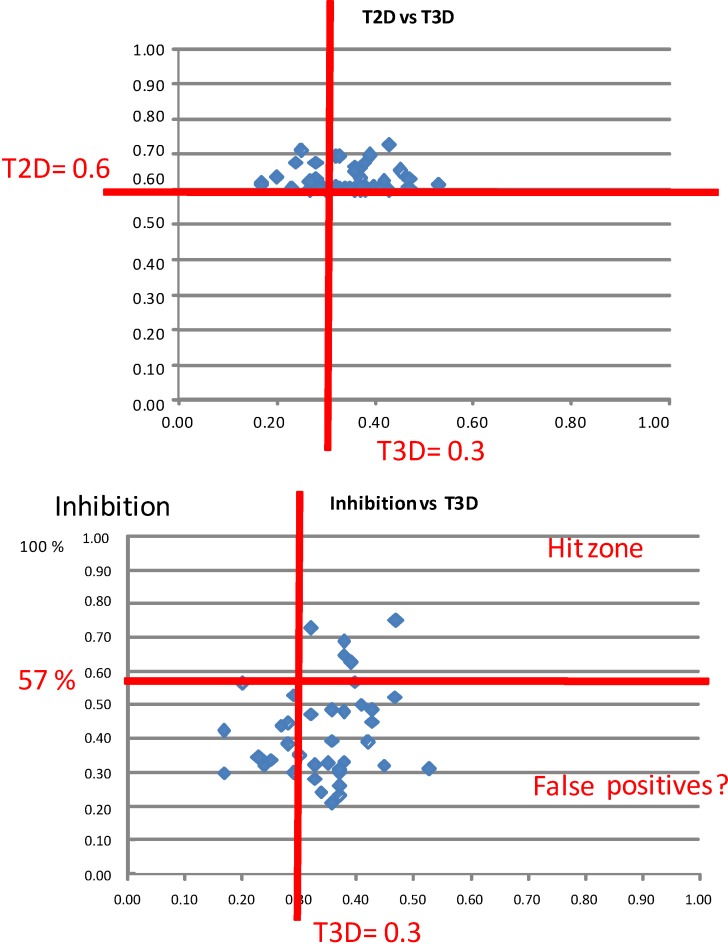
Scatter plot of all the measured compounds (41, active and not active) derived from the 3 seeds (#13,#18,#44) correlating the T2D and T3D similarity scores (**a**); and correlating the IC_50_ values and T3D values (**b**).

Figure 1a shows the scatter plot of correlating the 2D and 3D similarity scores of all the measured compounds (41, active and not active) derived from the three reference compounds (#13, #18, #44) in the first screening round. Since the five hits had T3D values > 0.3 we indicated such a value on Figure 1a. In Figure 1b PDE5 inhibition % values were correlated with the T3D values. Interestingly, the T3D values of the best hits were over 0.3 although together with lots of false positives (45% of all compounds measured). As an inhibition cut-off value, we applied 57% at 10 µM concentration (this inhibition % value provided a reasonable number compounds for concentration dependency studies).

**Figure 2:**
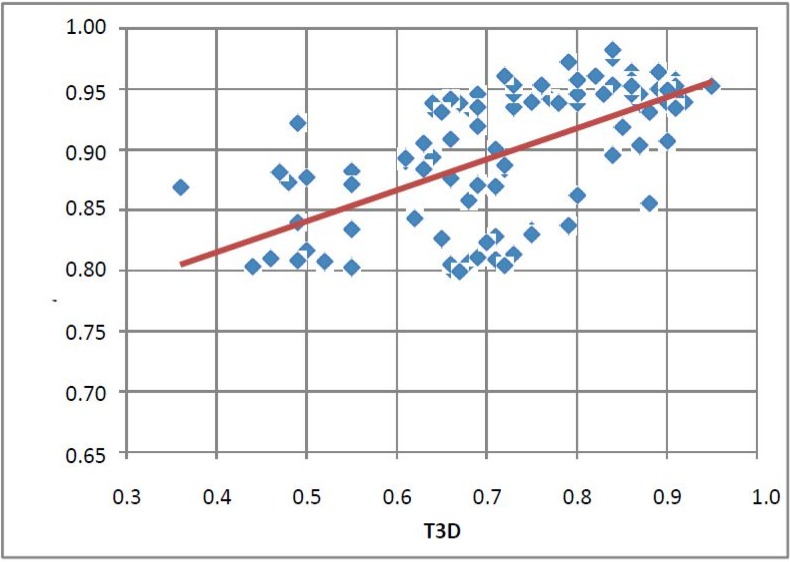
Correlation between T2D and T3D values of the compounds (104) selected for the second round screening.

The first round selection and screening led to the conclusion that T3D = 0.3 might have been a useful cut-off value. Based on the structure of the first round screening hits, 104 compounds were selected for the 2nd round (hit validation) screening by 2D methods as reported earlier [19]. The selection criterion was T2D ≥ 0.8. Now the 3D similarities of the selected set to the corresponding first round hit compounds were calculated and the T3D values were between 0.35 and 0.95. The 2D/3D correlation is shown in Figure 2. There is a certain trend that with increasing 2D values the 3D values are also increasing but in a lower extent. Interestingly, the majority of the compounds have T3D values above 0.6.

**Figure 3:**
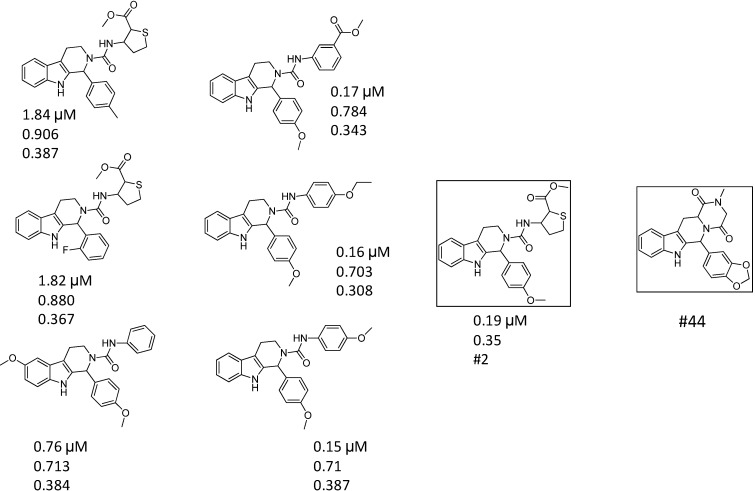
Hits derived from #44 seed with the T3D similarities to the first round hit and seed (reference compound)—Series #2.

**Figure 4:**
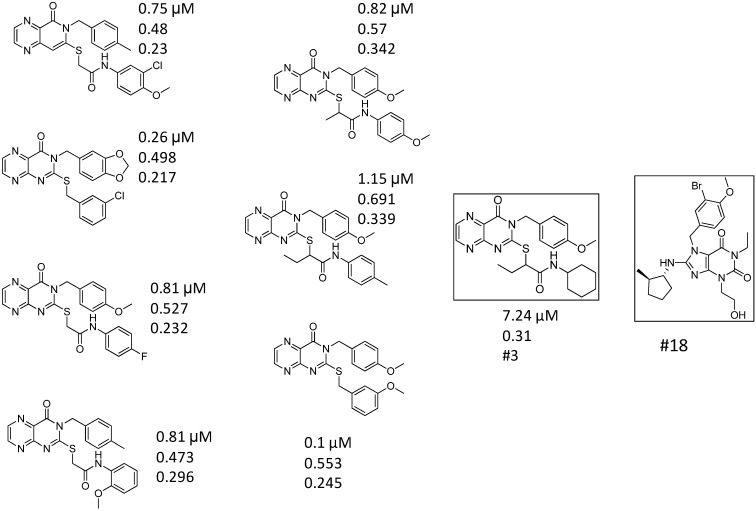
Hits derived from #18 seed with the T3D similarities to the first round hit and seed (reference compound)—Series #3.

Most of the second round hit compounds (13 out of 20) derived from two PDE5 reference compounds (#18, #44). We analyzed separately these two series of compounds. The 3D similarities between the 2nd round (IC_50_ ≤ 2 µM) and the first round hits as well as the reference compounds were displayed in Figure 3 and Figure 4. Figure 3 shows the IC_50_ values of compounds derived from #2 and #44 (upper value); the T3D values to #2 (middle) and the T3D values to the reference compounds #44 (lower value).

Similarly, Figure 4 shows the IC_50_ values of compounds derived from #3 and #18 (upper value); the T3D values to #3 (middle) and the T3D values to the reference compounds #18 (lower value). For those hit compounds the 2D similarities were above 0.8 by definition, 3D similarities to the corresponding first round hits were between 0.42 and 0.91, while to the corresponding original seeds varied between 0.22 and 0.34, respectively. Combining the T2D/T3D similarities into a single fusion score we found that T2D + T3D is ranging between 1.22 (0.8 + 0.42) and 1.71 (0.8 + 0.91) for the second round screening.

Analyzing the second round hit compounds (13) derived from #2 and #3 first round hits (Series #2 and Series #3 compounds) we got different results. While analogues in Series #2 have high T3D scores (between 0.7 and 0.9), those compounds that derive from Series #3 show significantly lower 3D similarity values (between 0.42 and 0.63) (Figure 3 and Figure 4) (note: 2D similarity is over 0.8 for all the compounds by definition). This clearly shows that such analogs could have significantly different binding characteristics than the first round hit they derive from. The significant improvement of the inhibitory activities confirms such findings (from 7.29 µM to 0.1 µM). We could also conclude that in Series #3 if the saturated ring (cyclohexyl) is replaced with aromatic moieties the activity is significantly increases.

Looking at the T3D values of these second round hits correlated with the original reference compounds (seeds) in Figure 3 and Figure 4 we could find again a major difference between the two series of compounds (Series #2 and #3). While analogues derived from #18 have higher T3D scores (between 0.31 and 0.39), those compounds that were derived from #3 show lower 3D similarity values (between 0.21 and 0.34).

Based on the above analysis we could conclude that T3D values are much more sensitive measures than their T2D counterparts and they reflect important structural features (e.g., conformational flexibility) that strongly depend on the structure. Interestingly, the T2D and T3D sensitivity is also structure dependent. We could also state that T3D = 0.3 would be a good cut-off value in the first round similarity selection and T3D = 0.5 or 0.6 in the second round (hit validation). Of course if we apply stricter filters the “focusing” would give probably a higher hit rate but some compounds (chemotypes) would have been lost. In summary, based on the above study we envisaged that combination of the 2D/3D similarity measures could have definite advantages in ligand-based virtual screening.

### 2.2. Case Study 2: Application of Combined 2D/3D Similarity Selection in Generating of a PDE4 Focused Library

In this second case study we applied Screen3D in a real virtual screening situation to select PDE4 inhibitor focused library, similarly from multi-million compound repositories. 

**Figure 5:**
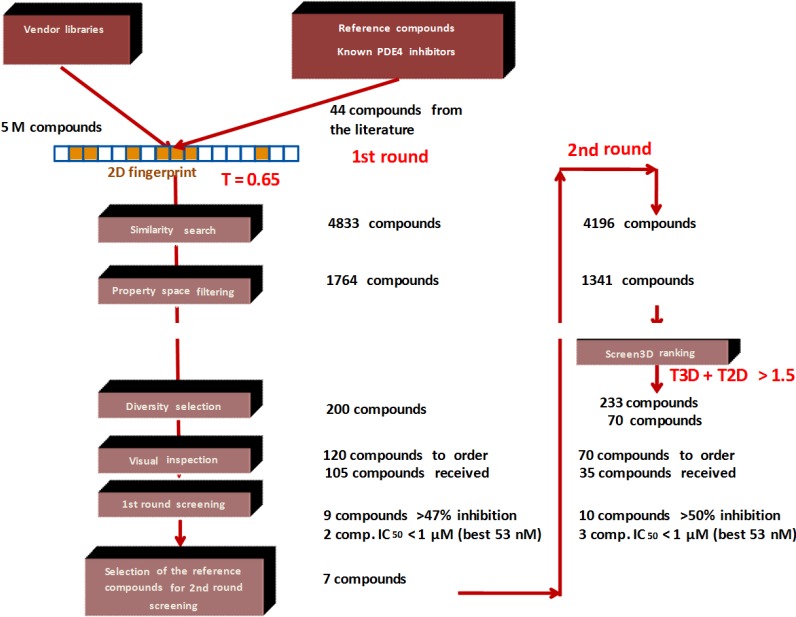
*In silico* selection scheme of the PDE4B focused library.

First we applied a similar 2D selection strategy as we reported for PDE5 [19]. In the second round (hit validation) library selection we used Screen3D in combination with the 2D approaches (Figure 5). The 2D similarity search in the first round was performed based on the structure of 44 known PDE4 inhibitors (collected from the literature) on our five million compound vendor collection. We selected PDE4 inhibitors as seed compounds that have low nanomolar inhibitory activities plus we added some interesting chemotypes, where no activities were reported in order to increase the chemical space. The similarity search was followed by property space filtering, applying the calculated ranges of 6 physico-chemical parameters (LogP, Mwt, tPSA, HBD, HBA and rotational bonds) relevant to drug-likeness with equal weight. Finally, simple diversity selection reduced the library size from 1764 to 200. After visual inspection of the compound set we send an inquiry for 120 compounds, and the available compounds (105) were ordered. *In vitro* measurements at one single concentration resulted in nine compounds (>47% inhibition at 10 µM concentration), and those were selected for measuring the concentration dependency determining the IC_50_ values. As a result seven compounds were considered as hits (IC_50_ ranges from 0.05 to 16 µM). The hit rate of the first round screening was 6.6% (>47% inhibition) and the success rate was 2.8% if we consider the hits below 2 µM (IC_50_). Table 2 shows the first round screening results (note: 47% inhibition was chosen instead of 50% to involve more compounds into the IC_50_ determination).

The seven first round hit compounds served as seed compounds for the second round (hit validation) library selection. Based on the experience we gathered during the analysis of PDE5 inhibitor library selection we decided to apply Screen3D as a filtering tool in combination with 2D similarity search at this stage. First we carried out a standard 2D similarity search since Screen3D could only handle a couple of thousand compounds rather than a couple of million. As a second step we calculated the 3D similarity values towards their corresponding first round hits for 1341 compounds that were obtained after 2D similarity search (T2D > 0.65) and the property space filtering.

**Figure 6:**
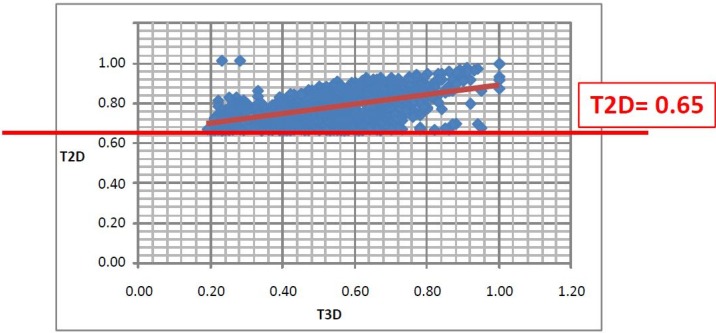
Correlation between the T2D and T3D values.

The 3D similarity values towards their appropriate first round hits for 1341 compounds were calculated and their correlation to the 2D score is shown in Figure 6). We found a similar trend as with PDE5 the 3D Tanimoto coefficient ranged from 0.19 to 0.99 while the 2D cut-off value was 0.65. We recognized again a weak linear correlation between the 2D and 3D similarity measures (Figure 6).

**Table 2:** 7 hits obtained in the first screening round.

ID	Structure	Inhibition	IC_50_ µM	T2D	Seed_ ID	Seed
**2**	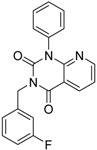	0.92	0.053	0.66	38	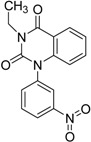
**3**	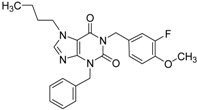	0.65	1.05	0.71	13	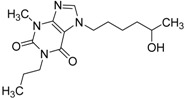
**5**	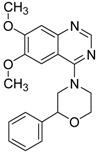	0.71	1.97	0.7	42	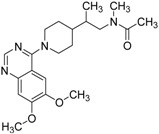
**4**	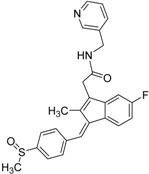	0.7	6.05	0.75	29	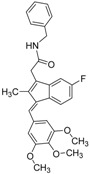
**1**	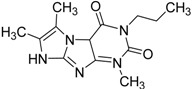	0.47	11.05	0.72	13	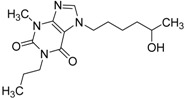
**6**	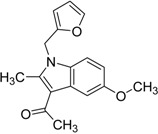	0.56	13.79	0.66	14	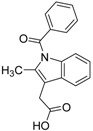
**7**	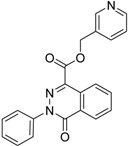	0.6	16.05	0.77	24	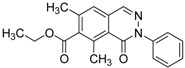

Based on earlier findings a combination of T2D and T3D looks more appropriate for reducing the number of compounds for biological screening and implementing conformational information. There are various options to combine those two scores. First, the T2D and T3D scores can be weighed differently giving more emphasis on any of the approaches. Secondly, the fusion score can be either an aggregation or a fraction.

There is an aggregation type of fusion score in 2D virtual screening which combines T2D similarity scores to more than one compound. In this approach first the similarity scores to “n” reference compounds are calculated for each compound within a medium sized compound library and then the “n” similarity measures obtained for each compound of the library are accumulated (“fused”) [20]. Furthermore, the cut-off values can also be debated. We propose a fusion score which treats the influence of T2D and T3D equally and aggregates the scores. A suitable cut-off value was proposed as T2D + T3D ≥ 1.5 based on the following considerations:

(1) Since T2D was ≥ 0.65 (cut-off value) at such relatively lower 2D similarity level only those compounds would be in the selection where the T3D similarity is high (>0.85). (*Note*: In the PDE5 study we found that the fusion score was between 1.22 and 1.7 in the 2nd round screening. Since in that case the T2D cut-off value was 0.8 low T3D values were also acceptable).
(2) Inversely, in the case of higher T2D values (max. 0.96) compounds could be selected even in the case of lower T3D values (min. 0.54).
(3) Our preference to equal weight of T2D and T3D scores can be explained by the objective of the hit validation, which requires relatively close (2D) analogues.

**Figure 7:**
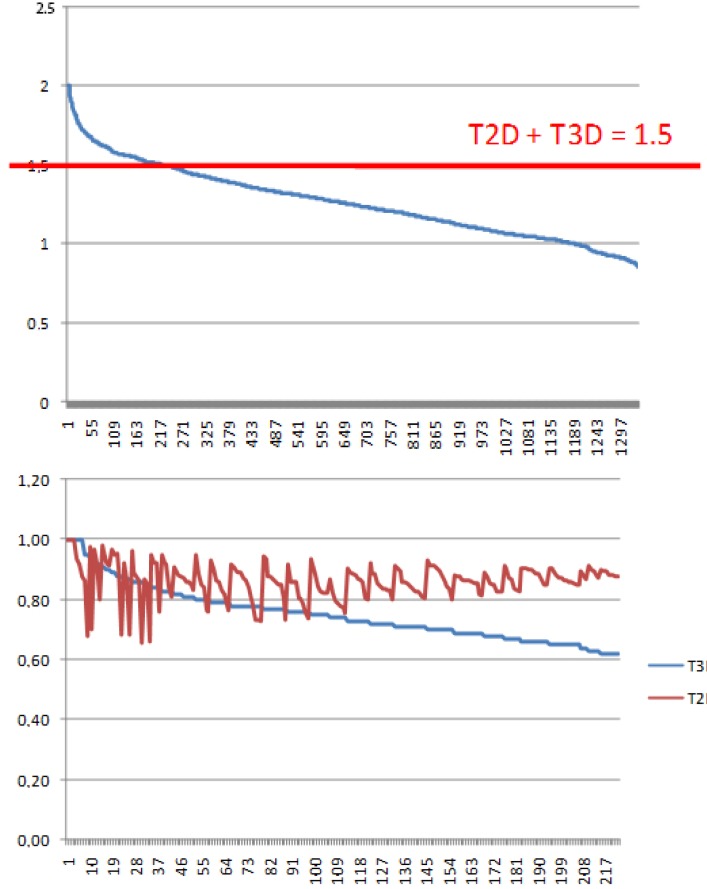
(**a**) Selection of 233 compounds out of 1341 that have a fusion score ≥ 1.5; (**b**) Increasing and fluctuating contribution of the 2D Tanimoto coefficient to the fusion score between 0.65 and 1, while 3D Tanimoto decreasing from 1 to 0.6 among the compounds that have a fusion score 1.5.

This combined cut-off value resulted in a relatively small number of compounds (from 1341 to 233) (Figure 7a) (nevertheless, 90 compounds were excluded that exceeded T3D = 0.65, due to the lower than 1.5 cumulative score). The representation of the compounds derived from the seven first round hits varied significantly showing that the T3D ranges and its distribution strongly depend on the chemotypes (see Table 3). For example analogues derived from #4 and #1 were recovered in the 233 set higher than 40% while other analogue groups were underrepresented (#3 and #6). This is partly due to the low T3D values (0.19 and 0.29 respectively).

**Table 3:** Distribution of the T3D values in the entire second round selection library and within the library having fusion score above 1.5.

ID	Structure	Analogues in the 1341 Set	Lowest T3D	Analogues in the 233 Set	Lowest T3D	Percentage in the 233 Set
**2**	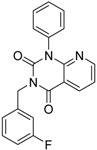	155	0.24	30	0.69	19.35
**3**	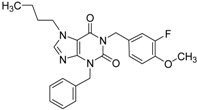	476	0.19	16	0.63	3.36
**5**	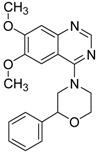	27	0.33	9	1.00	33.33
**4**	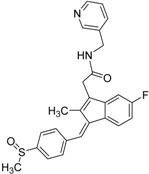	13	0.28	6	0.72	46.15
**1**	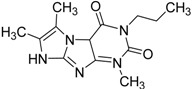	273	0.30	116	0.62	42.49
**6**	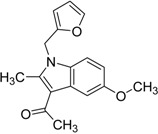	117	0.29	12	0.70	10.26
**7**	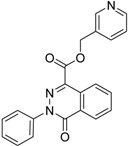	280	0.28	44	0.64	15.71

After the 2D/3D combination filtering, which resulted in 233 compounds, the library was reduced to 70 by diversity selection and this compound set was ordered. Unfortunately, only 35 compounds were available; however, the obtained compound set reflected the distribution of the initial set of compounds (233). *In vitro* screening of the small focused library resulted in 10 hits (IC_50_= 0.053–3.2 µM, Table 4). In general the contribution of T2D is gradually increasing but fluctuating between 0.65 and 1, while T3D is decreasing from 1 to 0.6 among the compounds that have a fusion score of 1.5 (Figure 7b).

**Table 4:** 10 hits obtained in the second screening round.

ID	Structure			Inhibition at 10 µM (%)	IC50 (µM)		T2D		T3D		Fusion Score			T2D/T3D	First Round Hit_ID			First Round Hits		Chemo Type		
**3**	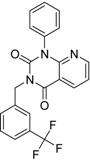			83	0.053		0.9		0.88		1.78			1.02	2			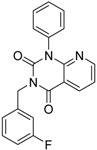		A2		
**5**	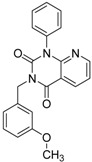			94	0.105		0.86		0.87		1.73			0.99	2			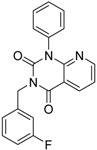		A2		
**4**	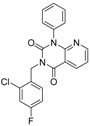			90	0.779		0.92		0.83		1.75			1.1	2			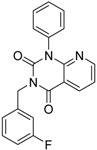		A2		
**6**	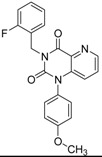			88	1.1		0.89		0.82		1.71			1.09	2			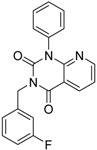		A2		
**8**	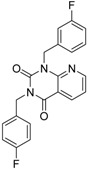			86	1.4		0.81		0.69		1.50			1.18	2			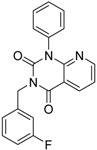		A2		
**7**	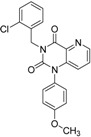			89	3.0		0.84		0.78		1.62			1.08	2			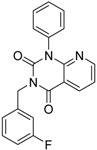		A2		
**1**	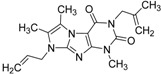			51	1.7		0.85		0.69		1.54			1.24	1			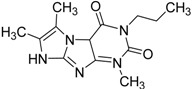		B1		
**2**	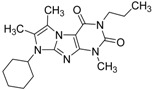			61	1.4		0.84		0.67		1.51			1.25	1			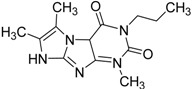		B1		
**9**	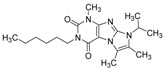			62	3.2		0.85		0.65		1.5			1.31	1			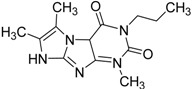		B1		
**10**	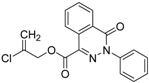			62	2.4		0.88		0.64		1.52			1.38	7			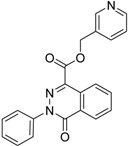		A11		

The hit rate of the 2nd round screening was 28.5% (>50% inhibition, at 10 µM), while 10% hit rate was calculated if only those compounds were considered that had an IC_50_ values < 2 µM (seven compounds).

**Figure 8:**
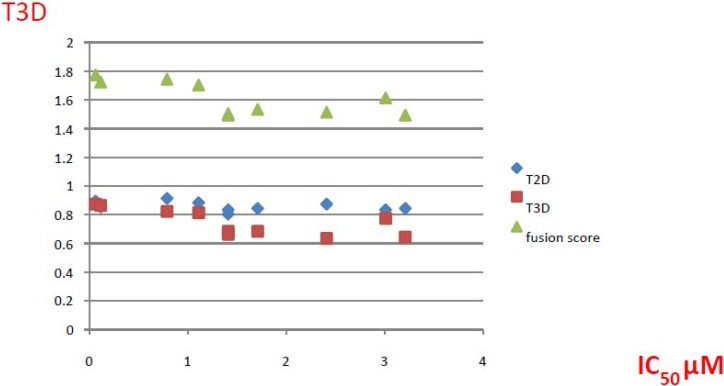
T2D/T3D and fusion score analysis of the 10 second round hits.

We also analyzed the correlation of the hits. The T2D/T3D correlation and fusion score analysis applied on the 10 second round hits revealed that six out of the 10 hits have a fusion score around 1.5, which is close to the cut-off value (Figure 8). It means that T3D values are apparently low in those cases while T2D is around 0.8. This finding suggests that the fusion score should be decreased to 1.3, which would allow the involvement of more compounds with lower T3D values. On the other hand it looks that the biological activity correlates well with the T3D values (which is actually more sensitive in nature).

#### 2.2.1. Structural Analysis of the Hits

**Figure 9:**
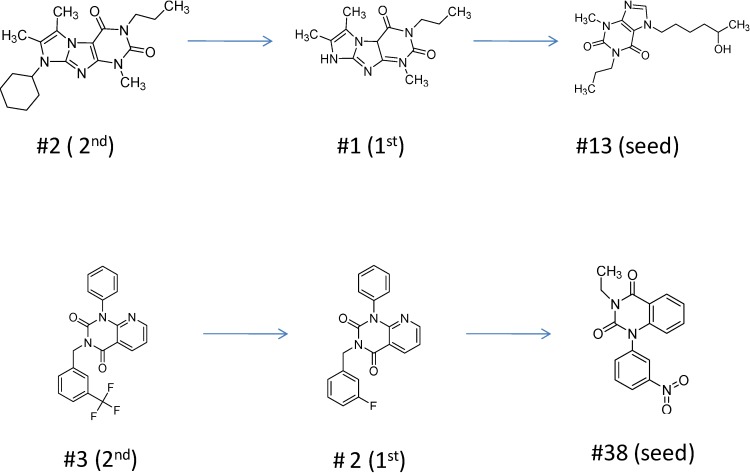
Hit evolution involving scaffold hopping. The hit evolution sequence of two second round hit compounds that involved scaffold hopping. #2 (2nd) → #1 (1st) → #13 (seed) (upper scheme) and #3 (2nd) → #2 (1st) → #38 (seed) (lower scheme).

Looking at the structural relationship of the first round hits and their seeds we could identify several pairs where the core structure is retained (3 → 13, 5 → 42, 4 → 29, 6 → 14, 7 → 24, Table 3) and others, where we could recognize scaffold hopping (1 → 13, 2 → 38) (Figure 9). In the second round screening only three chemotypes were represented in the selected seven compounds which derived from hit #1, #2 and #7 (Table 4). We searched PubChem for such chemotypes, whether they have already been reported as potential PDE inhibitors.

**Figure 10:**
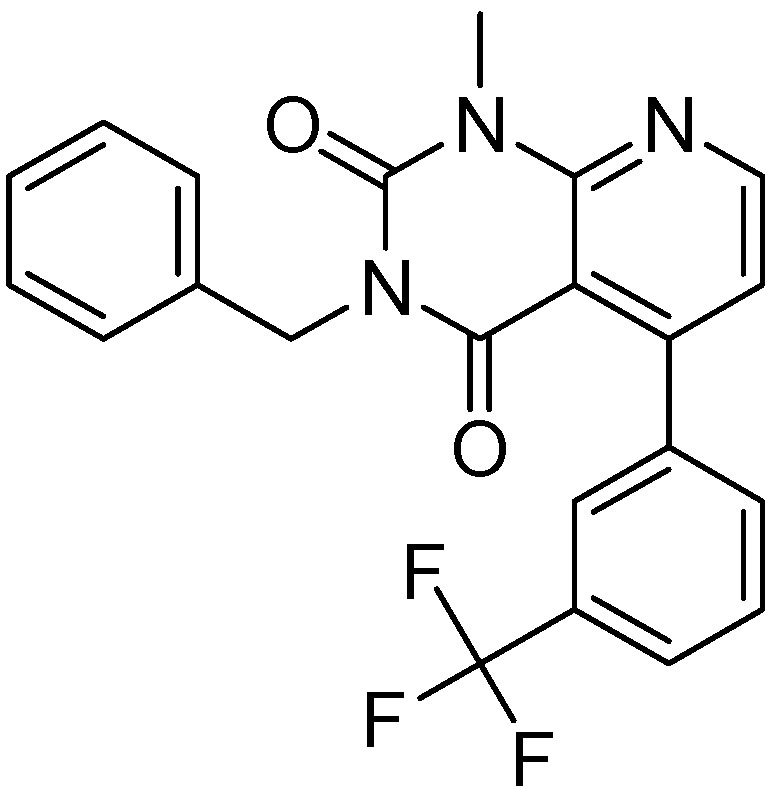
PubChem listed compound as analogue of the hit #3.

We found an analogue for hit #2 (1st round, 53 nM) what we might have missed during the search for reference compounds (Figure 10, same chemotype with different substitution pattern), however, the reported activity of such analogue was only 950 nM) [21].

Furthermore, for chemotypes that represented in hits #1 (1st round, 11 µM) and #2 (2nd round, 1.4 µM) we did not find any biological data in PubChem, the closest analogue exhibited biological activity as an adenosine A(1) and A(2A) antagonistic effect [22].

#### 2.2.2. PDE4B Selectivity

Recently our attention has turned to respiratory diseases mediated by PDEs. While PDE4B subtype is predominant in inflammatory cell regulation PDE4D inhibition has primarily CNS effects. Therefore, PDE4B is a therapeutic target for airway inflammatory diseases such as COPD while 4D is an anti-target and often responsible for side-effects, therefore certain selectivity for 4B against 4D would have additional values in the therapy [23]. Furthermore, multitarget therapies have become widely accepted in the recent years. Dual inhibition with either PDE3 or 7 and sometimes additional PDE5 inhibition had beneficial effects.

Although we did not discriminate PDE subtypes in the selection of the seed inhibitors thus we have not target selectivity in the study we decided to screen our hits compounds on the available PDE panel in order to determine the activity profile of our PDE4B hit compounds. Table 5 summarizes the results.

**Table 5:** The activity profile of 11 selected hit compounds (n.i. denotes no inhibition).

ID		Structure		PDE4B		PDE4B		PDE4D		PDE5		PDE2		PDE3		PDE7		PDE8		PDE9		PDE10A		PDE11		
				inh.% (10 µM)		IC50 (µM)		IC50 (µM)		IC50 (µM)		IC50 (µM)		IC50 (µM)		IC50 (µM)		IC50 (µM)		IC50 (µM)		IC50 (µM)		IC50 (µM)		
**3**		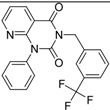		83		0.053		35.1		n.i.		n.i.		n.i.		n.i.		n.i.		n.i.		n.i.		n.i.		
**5**		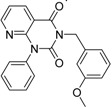		94		0.105		1.12		n.i.		n.i.		n.i.		n.i.		n.i.		n.i.		n.i.		n.i.		
**4**		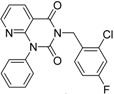		90		0.779		7.8		n.i.		n.i.		n.i.		n.i.		n.i.		n.i.		n.i.		n.i.		
**6**		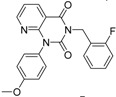		88		1.1		10.7		n.i.		n.i.		n.i.		n.i.		n.i.		n.i.		n.i.		n.i.		
**8**		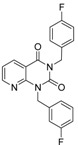		86		1.4		7.5		5.8		n.i.		n.i.		n.i.		n.i.		n.i.		n.i.		n.i.		
**7**		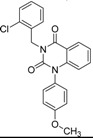		89		3.0		8		60		n.i.		n.i.		n.i.		n.i.		n.i.		n.i.		n.i.		
**2**		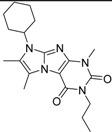		61		1.4		24		n.i.		n.i.		n.i.		n.i.		n.i.		n.i.		40.8		n.i.		
**1**		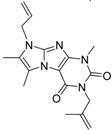		51		1.7		41		11.7		n.i.		n.i.		n.i.		n.i.		n.i.		2.4		40		
**9**		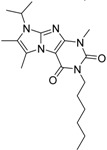		62		3.2		n.i.		12.5		n.i.		n.i.		n.i.		n.i.		n.i.		0.6		n.i.		
**11**		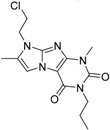		62		4.7		350		n.i.		n.i.		n.i.		n.i.		n.i.		n.i.		8.5		n.i.		
**10**		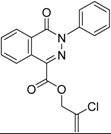		62		2.4		13.5		32.8		n.i.		n.i.		n.i.		n.i.		n.i.		n.i.		n.i.		

We found that most of the hit compounds showed some selectivity towards PDE4B and in some cases the selectivity was surprisingly high (50–80 fold) over PDE4D. Some of the hits inhibited PDE5 at low concentration which could be useful in the therapy while PDE10 inhibition could be avoided, since that target is more implicated in CNS disorders. The observed selectivity was further investigated by *in silico* methods in order to get into structural insights (discussed later).

#### 2.2.3. Structural Analysis of the Hit Compounds

**Figure 11:**
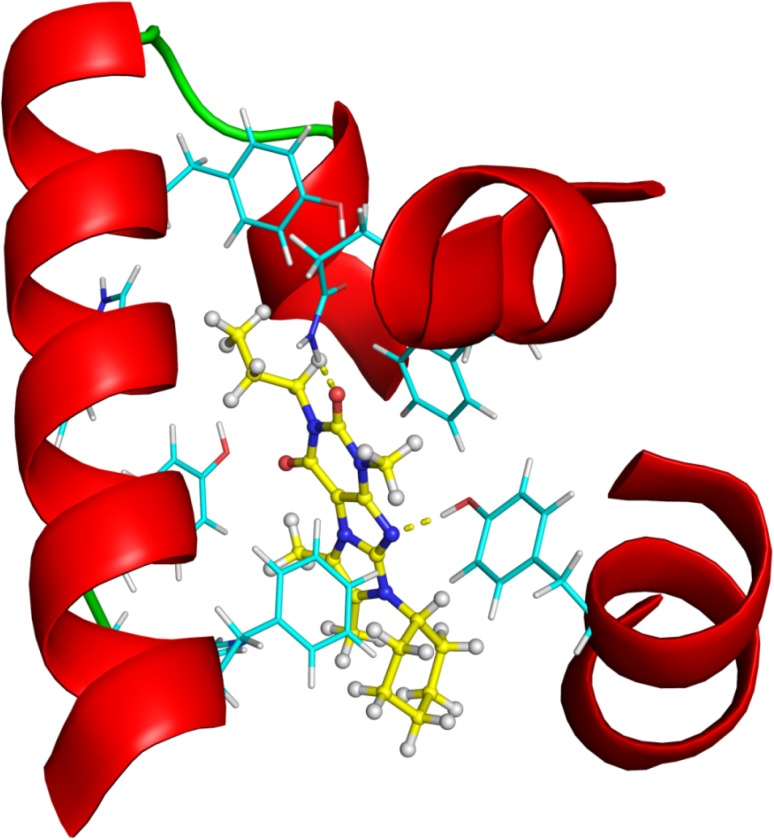
The structure of the Glide XP calculated PDE4B—#2 (2nd round hit) complex. Ligand carbon atoms are marked in yellow, while the nearest interacting protein residue side chains are shown in cyan color. The yellow dotted lines represent inter-molecular hydrogen bonds.

**Figure 12:**
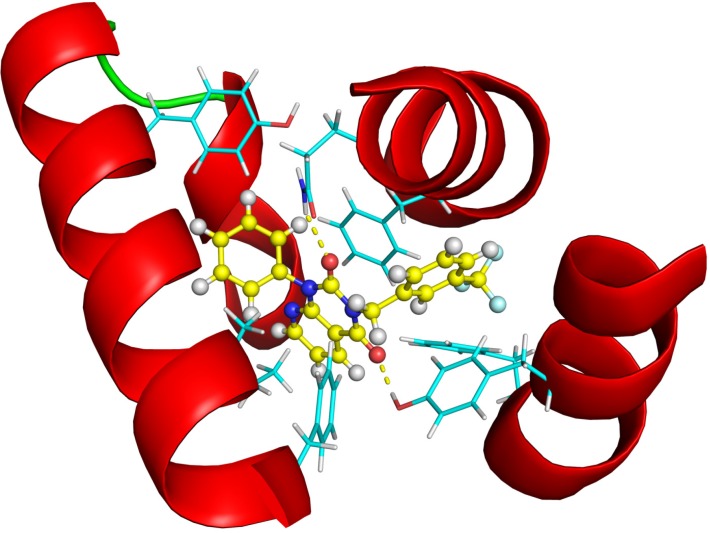
The structure of the Glide XP calculated PDE4B—#3 (2nd round hit) complex. Ligand carbon atoms are marked in yellow, while the nearest interacting protein residue side chains are shown in cyan color. The yellow dotted lines represent inter-molecular hydrogen bonds.

Since the X-ray structure of most PDEs is already known we carried out docking calculations on the best hits having an imidazo[1,2-g]purine-2,4-dione ring system (#2 hit—2nd round) and substituted 1*H*-purine-2,6-dione (#3 hit—2nd round). First we docked #2 and #3 into the PDE4B target structure in order to get information about the probable binding mode using the Glide XP program of the Schrodinger Suite 2013-3 package [24]. We used the 3G45 [25] structure from the PDB [26,27] database. The best scoring pose of ligand #2 and #3 (2nd round hits) can be seen in Figure 11 and Figure 12, respectively. Figures were created using the PyMOL [28] software.

**Figure 13:**
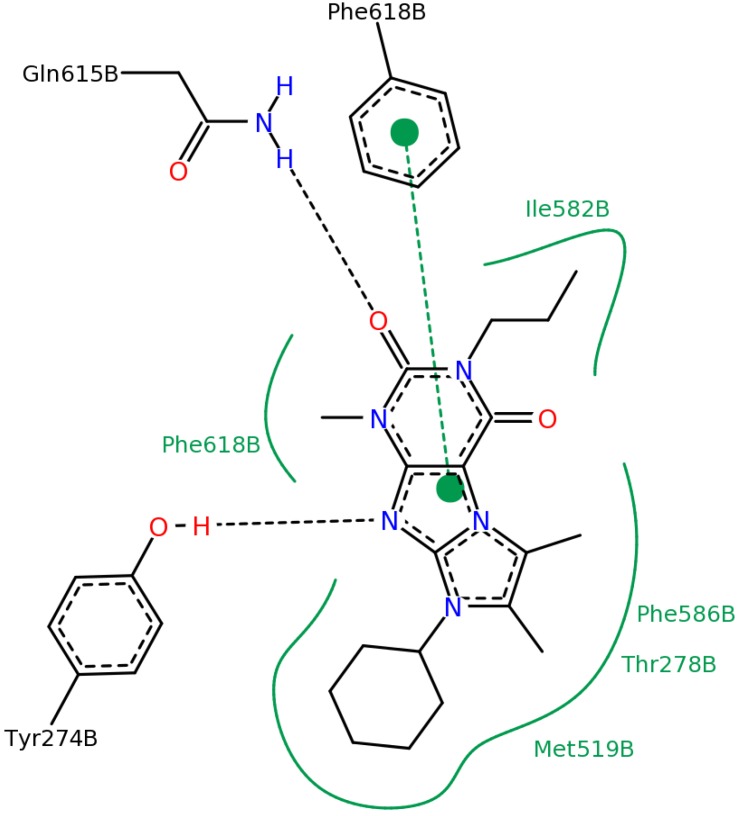
Diagram of the most important interactions in the Glide XP calculated PDE4B—#2 (2nd round hit) complex identified by PoseView. Black dashed lines represent hydrogen bonds, green solid lines represent hydrophobic interactions and green dashed lines represent aromatic interactions.

**Figure 14:**
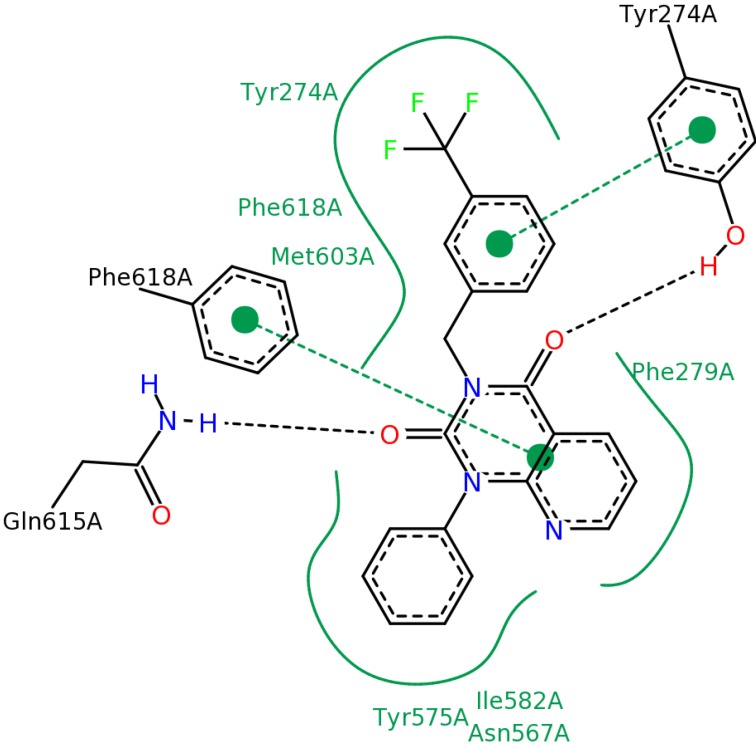
Diagram of the most important interactions in the Glide XP calculated PDE4B—#3 (2nd round hit) complex identified by PoseView. Black dashed lines represent hydrogen bonds, green solid lines represent hydrophobic interactions and green dashed lines represent aromatic interactions.

In order to demonstrate the most important interactions between the ligands and the binding site we created 2D structure diagrams of the ligand and its interacting residues using the PoseView [29] software. These diagrams can be seen in Figure 13 and Figure 14.

**Figure 15:**
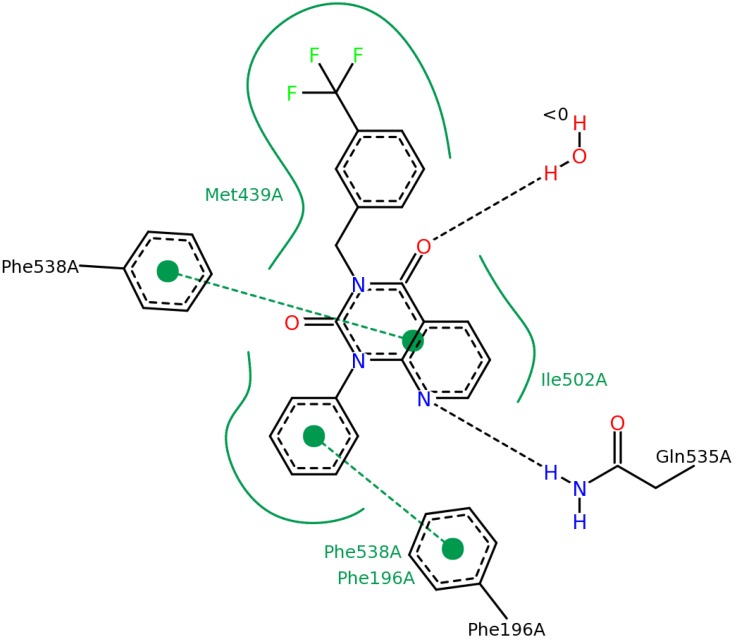
Diagram of the most important interactions in the Glide XP calculated PDE4D—#3 (2nd round hit) complex identified by PoseView. Black dashed lines represent hydrogen bonds, green solid lines represent hydrophobic interactions and green dashed lines represent aromatic interactions.

In the case of ligand #3 in Figure 14 we can see that the Tyr^274^ residue (according to the PDB numbering), belonging to the UCR2 domain of PDE4B, does not only contribute to the ligand binding through aromatic interaction but it also participates in the formation of a hydrogen bond. We docked ligand #3 (2nd round hit) into the 3G4G PDE4D structure. The PoseView diagram of this complex can be seen in Figure 15. The equivalent of the Tyr^274^ is the Phe^196^ residue in the PDE4D structure, which cannot participate in the formation of a hydrogen bond. This additional H-bond might explain the high PDE4B specificity of ligand #3 (2nd round hit, IC_50, PDE4B_ = 0.053 µM, IC_50, PDE4B_ = 35.1 µM).

In order to investigate the PDE subtype specificity we performed MM-GBSA binding energy prediction calculations on some hits. We used the X-ray structure of the longest available versions of PDE4B, PDE4D, PDE5A and PDE10A found in the PDB, namely the 3G45 [25], 3G4G [25], 2H42 [30] and 3HR1 [31] structure, respectively. The results of such calculations usually show good correlation with experimental results [32] (see Table 5 *vs.* Table 6) and the calculated binding energies obtained with the same receptor structure can be used for comparison to experimental binding affinities.

In order to get precise result Induced Fit Docking (IFD) calculations [33] were done in the case of 2 ligands using all receptor structures. During this procedure the binding site residues are allowed to change conformation in order to adapt to the ligand structure. In the best scoring complex structures MM-GBSA binding energy calculations were performed using the Prime module of the Schrödinger Suite.

The ranking based on the predicted binding energies shows nice correlation to the experimental IC_50_ values of these ligands. In all cases where no inhibition was detected experimentally the calculated ΔG_bind_ values are worse than −70 kcal/mol, these cases are marked in *italics* (Table 6). The binding energy and the inhibition data are particularly in good agreement for #11 since this compound also inhibits PDE10A at relatively low concentration (IC50 = 8.5 nM) beside PDE4B.

**Table 6:** ΔG_bind_ energies obtained from MM-GBSA calculation for various PDE variants.

ΔG_bind_ (kcal/mol)	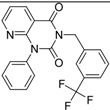 #3 in the 2nd round	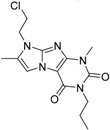 #11 in the 2nd round
PDE4B (3G45)	−113.367	−86.648
PDE4D (3G4G)	−103.014	−85.913
PDE5A (2H42)	−*68.768*	−*67.397*
PDE10A (3HR1)	−*69.916*	−92.082

#### 2.2.4. Investigation of Structure- Activity Relationships by 3D Modeling

**Figure 16:**
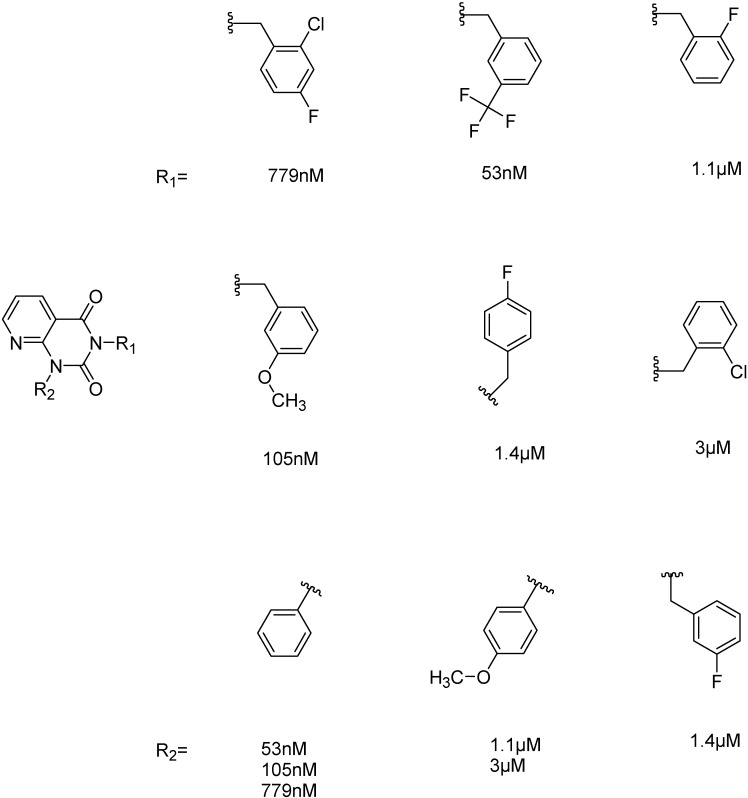
Preliminary structure-activity relationships of the most common chemotype of the 2nd round screening.

For the most common chemotype in our study (1*H*-purine-2,6-diones) we analyzed the various substitutions around the core in order to draw some conclusion regarding the structure—activity relationships. It is obvious that meta-substituted benzyls at R_1_ position are favored together with unsubstituted phenyl groups at R_2_ (Figure 16).

In order to model the observed structure activity relationships we performed MM-GBSA ΔG_bind_ calculations using the Prime module of the Schrödinger Suite on a series of ligands which share the same scaffold (#3, #5, #4, #6, #8, #7—Table 7) using the 3G45 PDB receptor structure. The calculated ΔG_bind_ values cannot be compared directly to the IC_50_ values, only to the pIC_50_ = −log (IC_50_) values. There is a nice correlation between the predicted binding energies and the pIC_50_ values, the correlation coefficient R^2^ = 0.49.

**Table 7:** ΔG_bind_ values obtained from MM-GBSA calculation and experimental IC50/pIC50 values for ligands with the same scaffold.

ID	Structure	PDE4B	PDE4B	pIC_50_	ΔG_bind_ (kcal/mol)
		inh.% (10 µM)	IC50 (µM)		
**3**	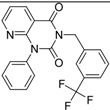	83	0.053	7.2757	−113.367
**5**	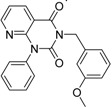	94	0.105	6.9788	−100.861
**4**	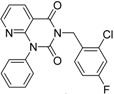	90	0.779	6.1085	−111.562
**6**	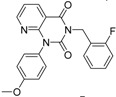	88	1.1	5.9586	−84.814
**8**	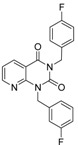	86	1.4	5.8539	−80.407
**7**	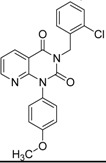	89	3	5.5229	−89.880

We calculated the binding energies with the MM-GBSA method for the #3 hit molecule and the co-crystallized ligand as a reference using the 3G45 PDB structure. The #3 hit molecule has more preferable predicted binding energy (ΔG_bind_^MM−GBSA^ = −113.367 kcal/mol) than the crystal ligand (ΔG_bind_^MM−GBSA^= −101.679 kcal/mol—8-(3-nitrophenyl)-6-(pyridin-4-ylmethyl)quinoline, PDE4B inhibition = 4.4 nM). Then we removed the UCR2 domain from the receptor structure and we repeated the calculation for ligand #3 and the crystal ligand and we got −76.045 and −80.120 kcal/mol, respectively. These calculations indicate that the UCR2 domain is required for high affinity binding of these ligands, like in the case of rolipram [34].

## 3. Experimental

### 3.1. Computational Methods

#### 3.1.1. 2D Similarity Search

For 2D similarity search, we applied fingerprints as implemented into InstJChem software (ChemAxon Ltd., Budapest, Hungary) [35]. A compound was defined as an analogue if the Tanimoto coefficient (Tc) [36] to any reference compound was >0.65. The searchable drug-like chemical space was composed by existing/real compounds: non-exclusive commercial libraries were available from the actual edition of the top vendor databases (~5 million compounds): ChemBridge, ChemDiv, Asinex, Enamine, IfLab, UkrOrg, AMRI, Specs, Maybridge, Interbioscreen [37,38,39,40,41,42,43,44,45,46]. The reference/active chemical space was defined by collecting known PDE inhibitors (“seeds”) from available literature, PubChem [2] and various commercial (annotated) databases including compounds with biological activity data from preclinical to clinical candidates and preferably with efficacies below 1 µM. As a second step the structures were clustered to major chemotypes and reviewed to remove the redundant structures.

#### 3.1.2. Calculation of the Physico-chemical Parameters and Property-based Filtering

Physicochemical descriptors are used in property–based drug design and can be used to refine the 2D similarity search [47,48]. The primary physicochemical parameters are Molecular weight, Log P, H-bond donors, H-bond acceptors, H-bond donors, Rotatable bonds, Topological polar surface area. The first 4 parameters are included in the Lipinski’s Rule of 5 [49], while the additional 2 are among the Veber rules [50]. Those rules propose certain general limits for library filtering, which correspond to a parameter “window” favorable for oral absorption. Target-specific parameter space (different from the general Lipinski and Veber rules) defined by the active reference compounds is practical for library filtering since various target families have a distinct property range as conclude by Morphy *et al*. [51] in his recent study.

The physico-chemical parameters (Mwt, LogP, H-bond donors/acceptors, rotatable bonds and topological polar surface area) were calculated by the calculation suit of InstJChem (ChemAxon Ltd.).

#### 3.1.3. 3D Ligand-Based Similarity Search for Ranking and Filtering of Focused Libraries

For 3D ligand-based similarity search we applied flexible alignment through the Match algorithm of the Screen3D software (Screen3D 5.1, ChemAxon Ltd. Budapest). Practically Match algorithm applies first a marking step of the specific atoms with predefined atom types and pharmacophore rules, which are followed by the calculations of minimum and maximum possible intramolecular distances between every atom-atom pair during the high-throughput continual conformational scan. Intramolecular distances are collected for the formation of distance range histograms. A single histogram represents the cumulative distance range distribution of the given type of atoms from the selected atoms. Then the distance ranges are coded in histograms and two histograms can be compared using histogram Tanimoto, where each histogram has two identifiers, an atom that is assigned to and an atom type. Finally, an atomic similarity score is calculated between a single atom of the query (a) and that of the target (b) using their histograms [52].

#### 3.1.4. Diversity Selection

In order to better/further reduce the combined selection to a reasonable and affordable library size, the above methods were complemented with diversity selection. Diversity selection was carried out by Similarity Manager (CompuDrug Int., Sedona, AZ, USA).

#### 3.1.5. 3D Visualization

The primary interactions between the PDE4B and the best hit compounds were demonstrated by using Schrodinger Suite 2013-3 package [24]. The predicted PDE4B ligand complex structures were visualized using the PyMol program [28]. Primary interactions between the PDE4b and the best hit compounds were demonstrated using the PoseView program [29].

#### 3.1.6. Ligand Docking

The protein structures were prepared using the Protein Preparation Wizard of the Schrodinger Suite 2013-3 [24] with the default settings. After preprocessing the hydrogen bonding network was optimized automatically and water molecules which have a distance of at least 5 Angstrom from hetero atoms or have less than 2 H-bonds to non-water atoms were removed. Metals were kept in all structures. Coordinates for missing side chain atoms were predicted automatically. The ligand structures were prepared with the Ligprep module using the default parameters, except that the maximum number of stereoisomers was set to 4. Default parameters were used in all Glide XP, Induced Fit Docking and Prime MM-GBSA ΔG_bind_ calculations.

### 3.2. Assay Development and Biological Screening

#### 3.2.1. PDE5A1

The *in vitro* biological assay we used is based on PDE5 cleavage of the phosphodiester bond of [^3^H]cGMP resulting in [^3^H]5'-GMP, which is further converted to [^3^H]guanosine by snake venom nucleotidase. Unreacted cGMP is removed by solid phase extraction using an ion exchange resin. [^3^H]guanosine content of the supernatant is determined by scintillation counting. The obtained signal is proportional to the amount of cleaved cGMP.

As an enzyme source recombinant full length PDE5A1 expressed in baculovirus-Sf9 system was used for the assay development. PDE5 inhibitors Dypridamole, Zaprinast and Sildenafil were used to validate the assay [19].

#### 3.2.2. PDE4B2

The *in vitro* biological assay is almost identical to that we used for PDE5 with the significant difference, that the substrate is [^3^H]cAMP (substrate concentration was 1 µM) instead of [^3^H]cGMP [19]. The recombinant PDE4B2 was used as enzyme source for assay development. The full length PDE4B2 gene was cloned based on sequence information deposited in Genbank (Accession number: NM_001037339) and the sequence was verified by DNA sequencing. The full length PDE4B2 was expressed using a bacolovirus-insect cell expression system. We used Sf9 cell line and pVL1393 transfer vector. Recombinant virus infected Sf 9 lysate was used as the enzyme source. As a first step the recombinant PDE4B2 was titrated—and the appropriate amount of the cell lysate for inhibition experiments was determined. In the second step a known PDE4 inhibitor (3,5-dimethyl-1-(3-nitrophenyl)-1H-pyrazole-4-carboxylic acid ethyl ester) was used to validate the assay.

For selectivity measurements PDE2A3, PDE3A, PDE4D4, PDE7A1, PDE8B1, PDE9A1, PDE10A1 and PDE11A4 were cloned and expressed in Sf9 cells similar to PDE5 and PDE4B2. In case of PDE3, PDE7, PDE8 and PDE4D4 the substrate was [^3^H]cAMP and for PDE2, PDE9, PDE10, and PDE11 we used [^3^H]cGMP as substrate, which were used universally at 1 µM concentration. The following known inhibitors were used in the assays: BAY-60-7550 (PDE2), trequinsin hydrochloride (PDE3, PDE8), BRL 50481 (PDE7), tadalafil (PDE5, PDE11), BAY-73-6691 (PDE9) and papaverine hydrochloride (PDE10).

## 4. Conclusions

In summary 3D shape/flexibility-based similarity search (Screen3D) is a sensitive and fast approach to select potentially active compounds [53]. It can be used either independently or in combination with 2D similarity search (depending on the size of the library). This method is particularly useful for refinement of 2D virtually prescreened libraries. Alternatively 2D/3D similarity selection methods can be combined. Thus, we proposed and applied a fusion (2D/3D) score and cut-off value. The novel fusion score approach led to improved hit rate and identification of novel chemotypes and significantly active compounds within known chemotypes. Since T3D is rather chemotype selective the fusion score can be applied differently according to individual chemical classes rather than an ultimate score will be used. Further refinement and investigation is needed to device a fusion score which is less sensitive to structural differences. 

## Supplementary Materials

Supplementary materials can be accessed at: http://www.mdpi.com/1420-3049/19/6/7008/s1.
