# Supplementary material for: Combination of 2D/3D Ligand-Based Similarity Search in Rapid Virtual Screening from Multimillion Compound Repositories. Selection and Biological Evaluation of Potential PDE4 and PDE5 Inhibitors
Source: Molecules. 2014 May 28;19(6):7008–39. doi: 10.3390/molecules19067008 (PMC6270928; doi:10.3390/molecules19067008)
Supplement: Supplementary file 1 [file molecules-19-07008-s001.pdf]

Supplementary Materials

Table S1. PDE5 inhibitor reference compounds (**44**).

PDE5 reference comounds			
**ID**	**Structure**	**Name**	**CAS number**
**1**	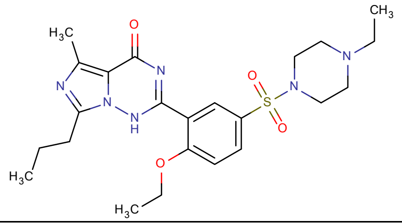	verdanafil	224785-90-4
**2**	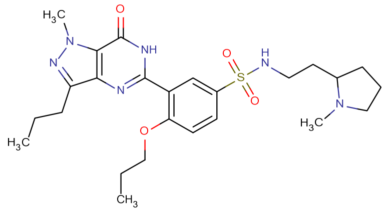	Udenafil	268203-93-6
**3**	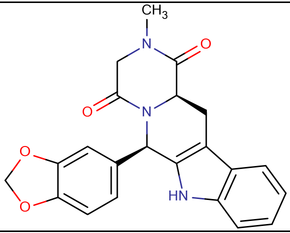	Tadalafil	171596-29-5
**4**	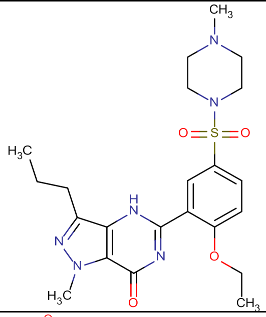	Sildenafil	139755-83-2
**5**	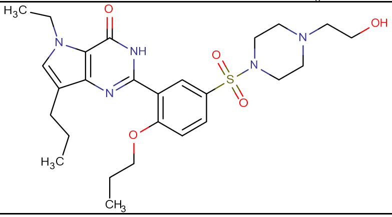		
**6**	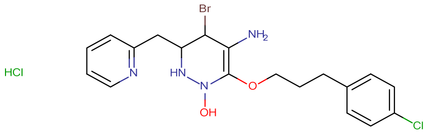		
**7**	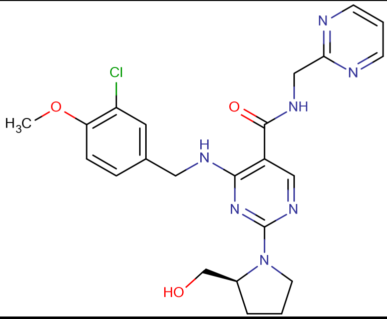	Avanafil	330784-47-9

Table S1. *Cont.*

PDE5 reference comounds			
**ID**	**Structure**	**Name**	**CAS number**
**8**	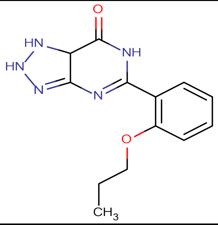	Zaprinast	37762-06-4
**9**	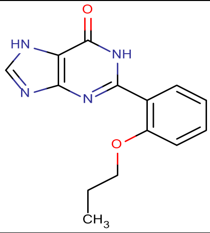		
**10**	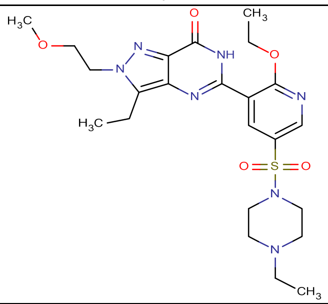	gisendafil	334826-98-1
**11**	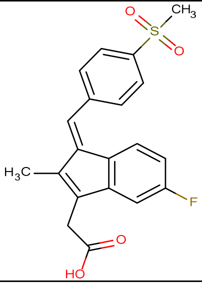	sulindac sulfon	
**12**	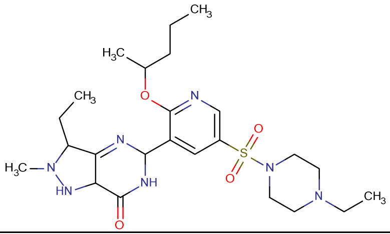		
**13**	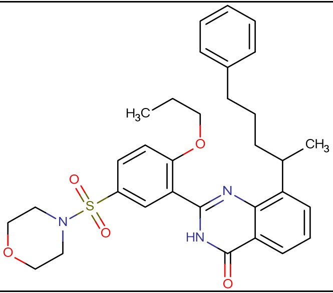		
**14**	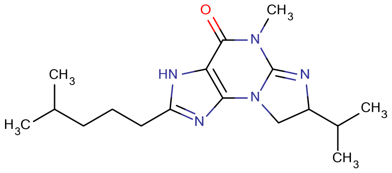		

Table S1. *Cont.*

PDE5 reference comounds			
**ID**	**Structure**	**Name**	**CAS number**
**15**	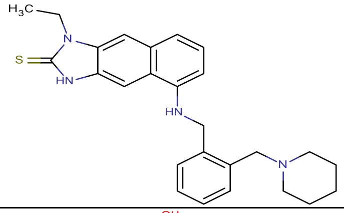		
**16**	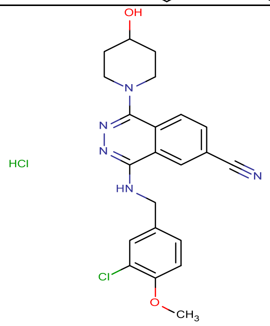		
**17**	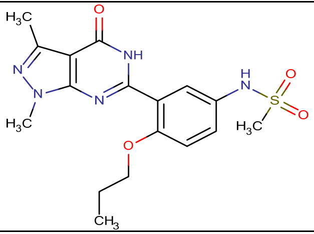		
**18**	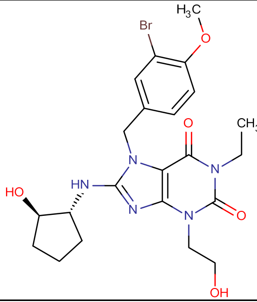	Desantafil	569351-91-3
**19**	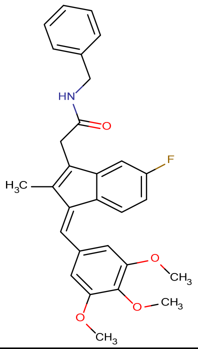		200803-37-8
**20**	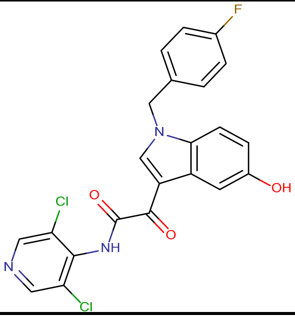		257892-33-4

Table S1. *Cont.*

PDE5 reference comounds			
**ID**	**Structure**	**Name**	**CAS number**
**21**	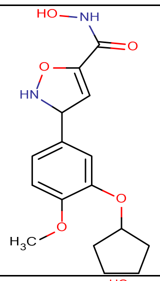		223919-71-9
**22**	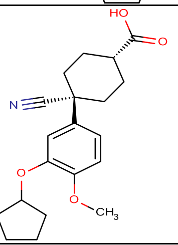	Ariflo; Cilomilast; SB 207499	153259-65-5
**23**	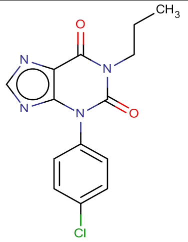	Arofylline	136145-07-8
**24**	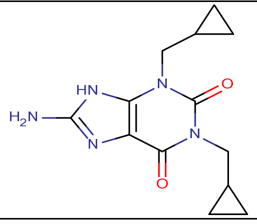	Cipamfylline	132210-43-6
**25**	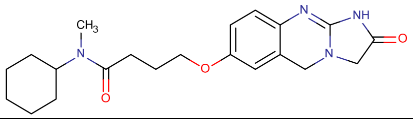	Lixazinone	94192-59-3
**26**	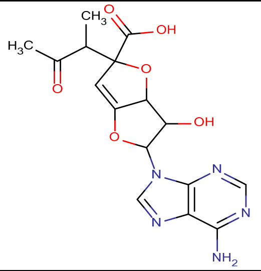		79030-08-3
**27**	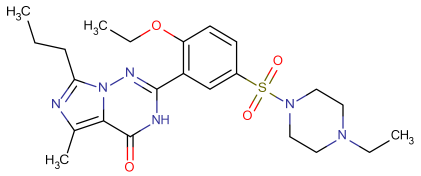		
**28**	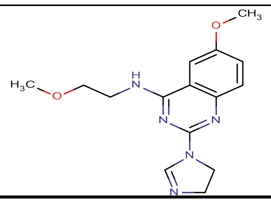		

Table S1. *Cont.*

**PDE5 reference comounds**			
**ID**	**Structure**	**Name**	**CAS number**
**29**	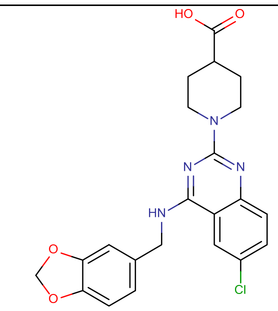		
**30**	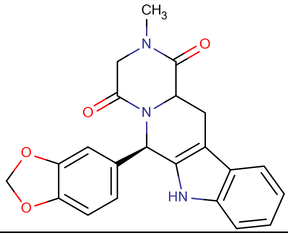		
**31**	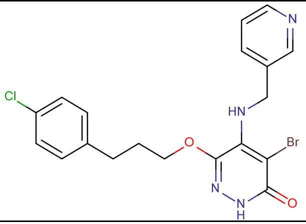		
**32**	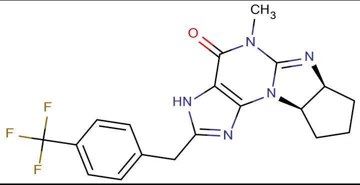		
**33**	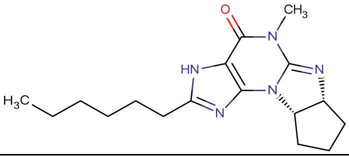		
**34**	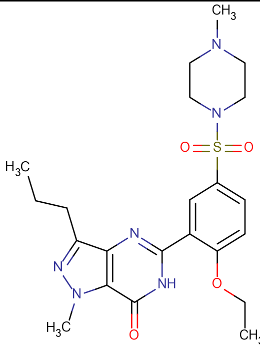		
**35**	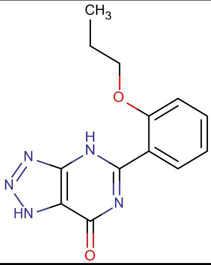		

Table S1. *Cont.*

PDE5 reference comounds			
**ID**	**Structure**	**Name**	**CAS number**
**36**	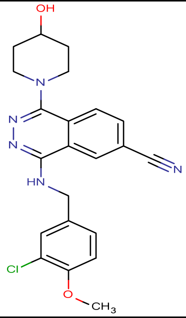		
**37**	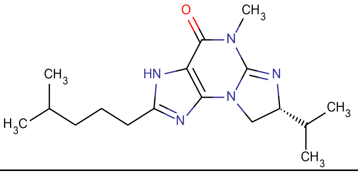		
**38**	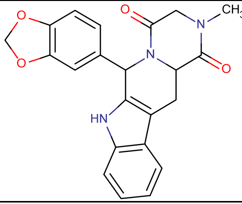		
**39**	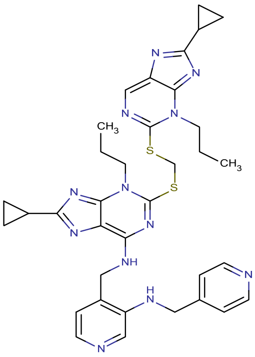		
**40**	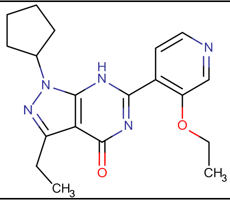		
**41**	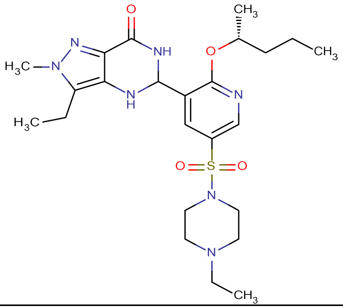		
**42**	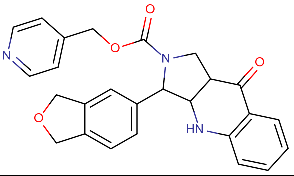		

Table S1. *Cont.*

PDE5 reference comounds			
**ID**	**Structure**	**Name**	**CAS number**
**43**	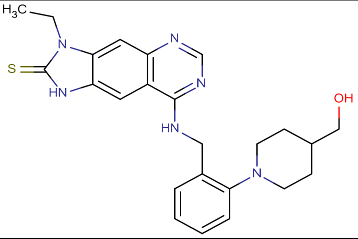		
**44**	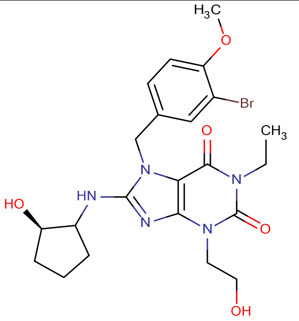		

Table S2. PDE4 inhibitor reference compounds (**44**).

PDE4 reference compounds	
**ID**	**Structure**
**1**	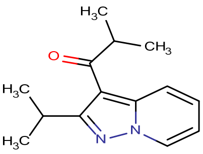
**2**	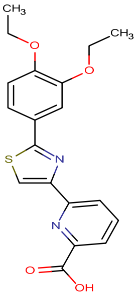
**3**	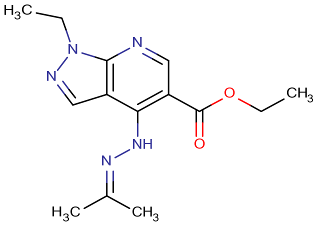
**4**	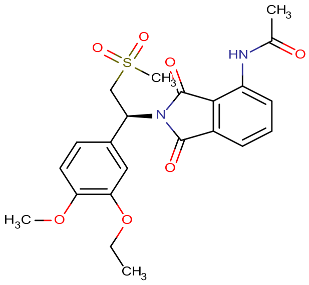

Table S2. *Cont.*

PDE4 reference compounds	
**ID**	**Structure**
**5**	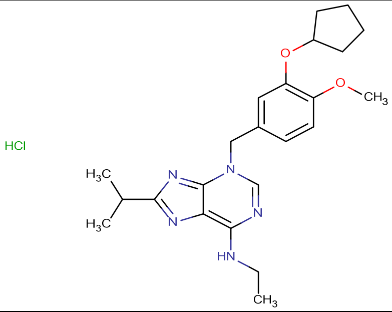
**6**	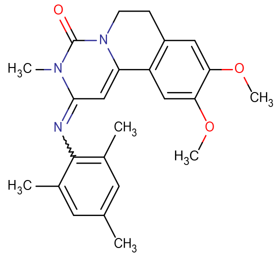
**7**	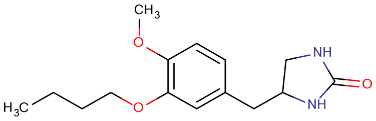
**8**	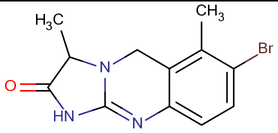
**9**	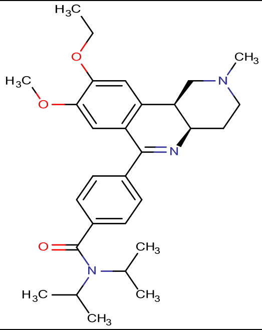
**10**	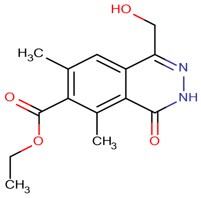
**11**	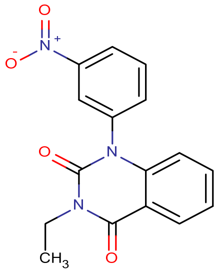

Table S2. *Cont.*

PDE4 reference compounds	
**ID**	**Structure**
**12**	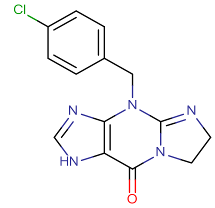
**13**	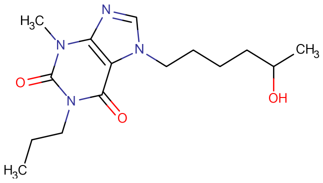
**14**	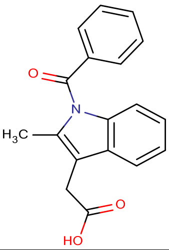
**15**	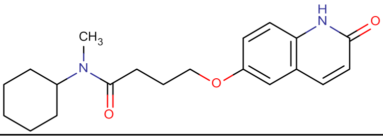
**16**	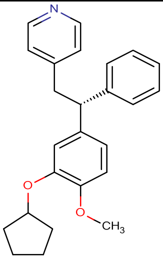
**17**	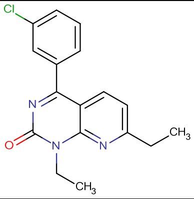
**18**	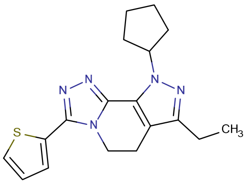
**19**	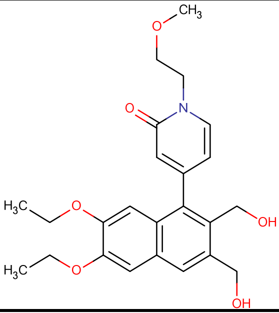

Table S2. *Cont.*

PDE4 reference compounds	
**ID**	**Structure**
**20**	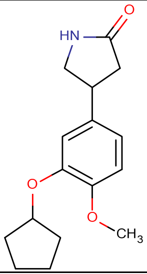
**21**	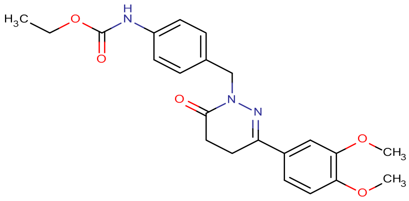
**22**	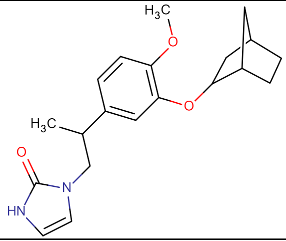
**23**	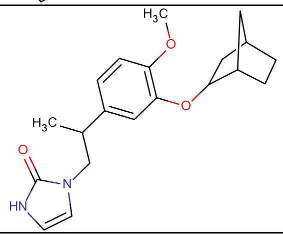
**24**	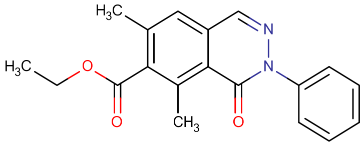
**25**	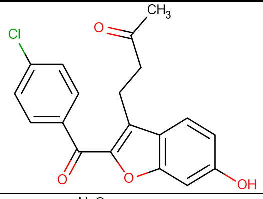
**26**	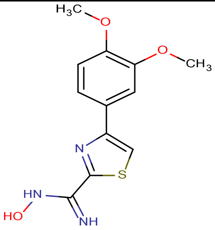
**27**	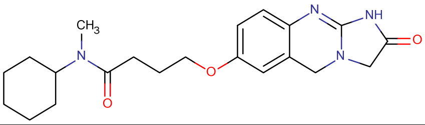

Table S2. *Cont.*

PDE4 reference compounds	
**ID**	**Structure**
**28**	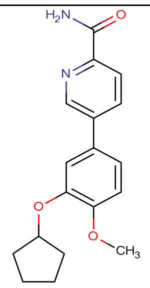
**29**	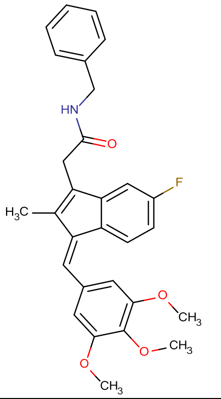
**30**	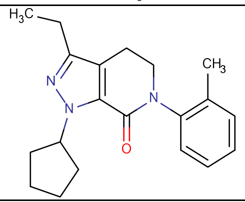
**31**	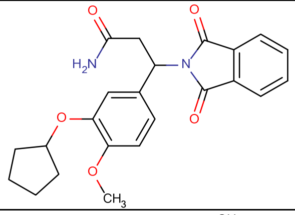
**32**	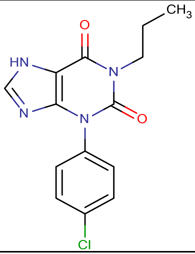
**33**	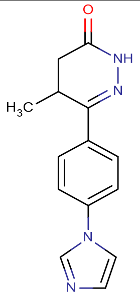

Table S2. *Cont.*

PDE4 reference compounds	
**ID**	**Structure**
**34**	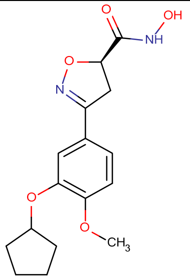
**35**	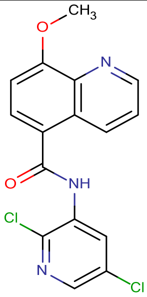
**36**	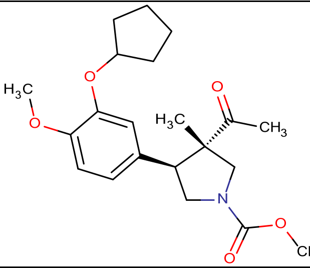
**37**	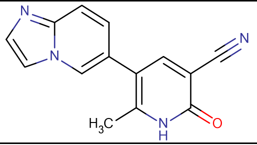
**38**	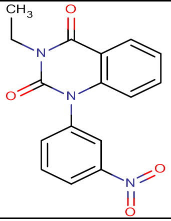
**39**	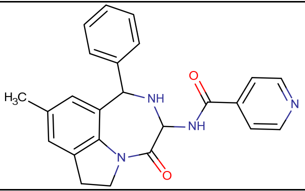
**40**	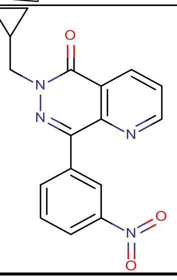

Table S2. *Cont.*

PDE4 reference compounds	
**ID**	**Structure**
**41**	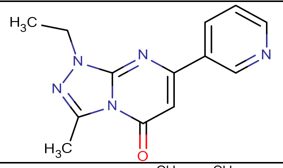
**42**	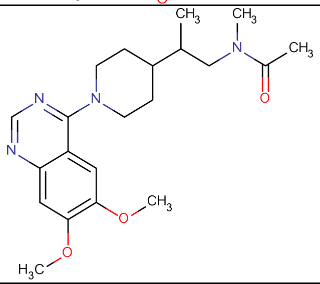
**43**	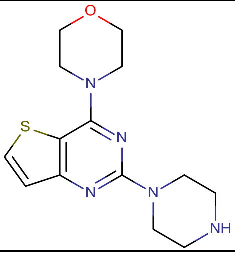
**44**	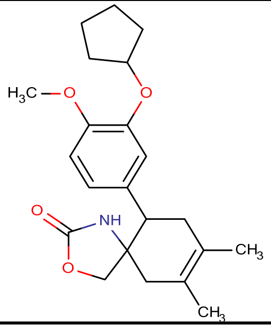
